# Comparison of Bupivacaine Trocar Site Infiltration and Peritoneal Instillation for Postoperative Pain in Pediatric Laparoscopic Surgery

**DOI:** 10.7759/cureus.79676

**Published:** 2025-02-26

**Authors:** Muhammad Mudasir Saleem, Mishal Pervaiz, Uswah Shoaib, Ismail Mazhar, Gull Sher, Sehar Khauteja Khan, Muhammad Ibrahim Tahir, Shahmir Ahmad Khan

**Affiliations:** 1 Pediatrics and General Surgery, Combined Military Hospital, Lahore, Lahore, PAK; 2 Anesthesiology, Combined Military Hospital, Lahore, Lahore, PAK; 3 Pediatric Surgery, CMH (Combined Military Hospital) Medical College and Institute of Dentistry, Lahore, PAK; 4 Internal Medicine, CMH (Combined Military Hospital) Medical College and Institute of Dentistry, Lahore, PAK; 5 Pediatric Surgery, Combined Military Hospital, Lahore, Lahore, PAK; 6 General Surgery, Combined Military Hospital, Lahore, Lahore, PAK; 7 Internal Medicine, Lahore Medical and Dental College, Lahore, PAK

**Keywords:** bupivacaine, intraperitoneal, laproscopic surgery, pain, trocar

## Abstract

Background

Laparoscopic surgery has become the treatment of choice for pediatric patients due to its numerous advantages over open surgery. However, postoperative pain remains a significant barrier to rapid recovery. The intraperitoneal use of local anesthetics for pain management is underutilized in the pediatric population. This study aimed to compare the effectiveness of bupivacaine administered at the trocar site versus in the peritoneal cavity for managing postoperative pain in pediatric laparoscopic surgery.

Methodology

This quasi-experimental study was conducted in the Department of Pediatric Surgery at the Combined Military Hospital, Lahore. A total of 80 patients who underwent laparoscopic surgery were divided into two groups of 40 each. The W Group received bupivacaine trocar site infiltration, while the P Group received intraperitoneal bupivacaine instillation at the end of surgery. Pain scores were recorded in both groups at one, two, four, six, and eight hours postoperatively. The need for rescue analgesia and the incidence of shoulder pain were also evaluated as secondary outcomes in both groups.

Results

The mean pain score was 19.15 ± 3.8 in the W group, compared to 17.30 ± 4.0 in the P group, with a calculated p-value of 0.03, indicating a significant difference between the two groups. Similarly, interval pain scores, the need for rescue analgesia, and the incidence of shoulder pain were significantly lower in the P group compared to the W group.

Conclusion

We concluded that intraperitoneal instillation of bupivacaine is more effective than trocar site infiltration with the same agent in reducing postoperative pain across various pediatric laparoscopic procedures. This approach should be adopted to maximize the benefits of minimally invasive surgery in the pediatric population.

## Introduction

Minimally invasive surgery has emerged as the treatment modality of choice in the pediatric population over the last decade and is being increasingly utilized across almost all fields of pediatric surgery [1]. It offers clear advantages over conventional open surgery, such as faster recovery, shorter hospital stays, earlier mobility, and a lower incidence of wound-related complications [2,3]. These benefits contribute to reducing the overall cost of treatment, which is particularly significant for developing countries with limited medical resources, such as Pakistan. However, pediatric laparoscopic surgery requires specialized short instruments, has a steep learning curve due to the precision needed in a confined space, and demands an in-depth understanding of the effects of pneumoperitoneum on the body for effective performance [4]. A wide range of simple and complex pediatric surgical procedures are now increasingly being performed using laparoscopy worldwide [5].

The etiology of pain following laparoscopic surgery is distinct and unique compared to open surgery. It includes pain from peritoneal irritation, referred shoulder pain caused by diaphragmatic irritation, and the incisional and visceral pain elements shared with open surgery [6]. Pain is considered the fifth vital sign, and its presence in pediatric patients is particularly challenging due to their limited ability to comprehend and communicate it effectively [7]. Consequently, multimodal analgesia, employing various analgesic drugs and different routes of administration, is deemed essential to prevent or reduce pain severity during the postoperative period [8].

The objective of this study was to compare the effectiveness of bupivacaine incision infiltration with peritoneal instillation in managing postoperative pain in pediatric laparoscopic surgery.

## Materials and methods

This was a quasi-experimental study conducted in the Department of Pediatric Surgery, Combined Military Hospital, Lahore, Pakistan, from May 2024 to October 2024. The study was approved by the Research Institutional Review Board Combined Military Hospital Lahore (approval number: 574/2024). 

Inclusion and exclusion criteria

Children aged 6-12 years, classified as American Society of Anesthesiologists (ASA) Physical Status I and scheduled for laparoscopic surgery, were included in the study. Children with severe cardiac issues, bleeding disorders, gross peritoneal contamination due to viscus perforation, a history of previous abdominal surgeries, or hypersensitivity to bupivacaine were excluded.

Sample size calculation

The WHO sample size calculator was used to determine the sample size, assuming a 40% incidence of postoperative pain in the pediatric population, an anticipated population proportion of P=0.40 [9], an absolute precision of d=0.10, and a 90% confidence level (CI). A total of 80 patients were included and divided into two groups of 40 each using the consecutive sampling technique. Group W received bupivacaine local infiltration, while Group P received bupivacaine intraperitoneal instillation following laparoscopic surgery.

Procedure

All patients underwent a thorough evaluation, including a detailed history and general physical examination. Relevant investigations such as ultrasonography of the inguinoscrotal region or abdomen were performed to confirm the diagnosis. A complete blood count (CBC) and hepatitis serology were obtained for anesthesia evaluation. Patients were kept nil per os (NPO) for eight hours for solids and two hours for clear liquids before surgery. Parents were counseled about the procedure and informed written consent was obtained.

During the induction of anesthesia, prophylactic antibiotics in the form of a single dose of third-generation cephalosporin (30 mg/kg) and nalbuphine (0.1 mg/kg), due to its opioid-sparing effects and lower risk of respiratory depression compared to other opioids, were administered. Postoperatively, acetaminophen was used as rescue analgesia to further minimize the risk of respiratory depression. All surgeries were performed under sevoflurane and propofol-based general anesthesia, maintaining inflation pressures of 8-10 mmHg.

After surgery, patients in Group W received local wound infiltration at all three trocar sites, while patients in Group P received intraperitoneal instillation at the surgical site. In both groups, 0.5% bupivacaine was diluted with 8-10 mL of distilled water and administered at a dose of 2 mg/kg body weight. [10]. Following recovery from anesthesia, patients were transferred to the recovery unit until fully awake.

**Figure 1:**
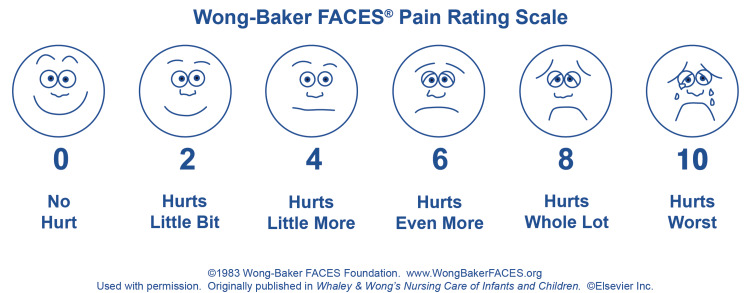
Wong-Baker FACES® Pain Rating Scale Image Source: Wong-Baker FACES Foundation [11]; used with permission

Postoperative pain was assessed using the FACES® Pain Rating Scale (Wong-Baker FACES Foundation, Oklahoma, United States) at one, two, four, six, and eight hours, evaluating overall pain perception in all patients, irrespective of surgical site variations [11], due to its child-friendly design, ease of understanding, and reliability in pediatric populations, which grades pain as: 0 = no hurt, 2 = hurts a little bit, 4 = hurts a little more, 6 = hurts even more, 8 = hurts a whole lot, and 10 = the worst hurt (Figure 1). A score greater than 6 at any time was classified as severe pain, and rescue analgesia was administered as intravenous acetaminophen (10 mg/kg body weight). If the pain score persisted above 6 after rescue analgesia, oral ibuprofen was given. The FACES Scale was explained to the patients so that they understood and were able to choose the face that best illustrated the physical pain they were experiencing. It was used by the patients directly.

Shoulder pain was assessed at all measured intervals by asking patients to respond with "yes" or "no." The need for rescue analgesia and the presence of shoulder pain were recorded as secondary outcomes.

Statistical analysis

The IBM SPSS Statistics for Windows, Version 24.0 (2016; IBM Corp., Armonk, New York, United States), was used for data analysis. Quantitative variables, such as age and pain scores, were presented as mean ± standard deviation. Qualitative variables, including gender, the need for rescue analgesia, and the incidence of shoulder pain, were expressed as frequencies and percentages. An independent sample t-test was employed to compare variables, and a p-value of <0.05 was considered statistically significant.

## Results

A total of 80 patients were included in the study and divided into two groups of 40 each. Group W comprised 27 male (67.5%) and 13 female (32.5%) patients, while Group P consisted of 24 male (60.0%) and 16 female (40.0%) patients. The study included both upper and lower abdominal procedures due to the limited availability of pediatric laparoscopic cases in our setting. The mean ± standard deviation (SD) age in months was 104.97 ± 20.1 in Group W and 101.62 ± 19.4 in Group P. The p-value of 0.45 indicated no statistically significant difference in age distribution between the two groups, ensuring comparability in the use of the FACES Pain Scale.

**Figure 2:**
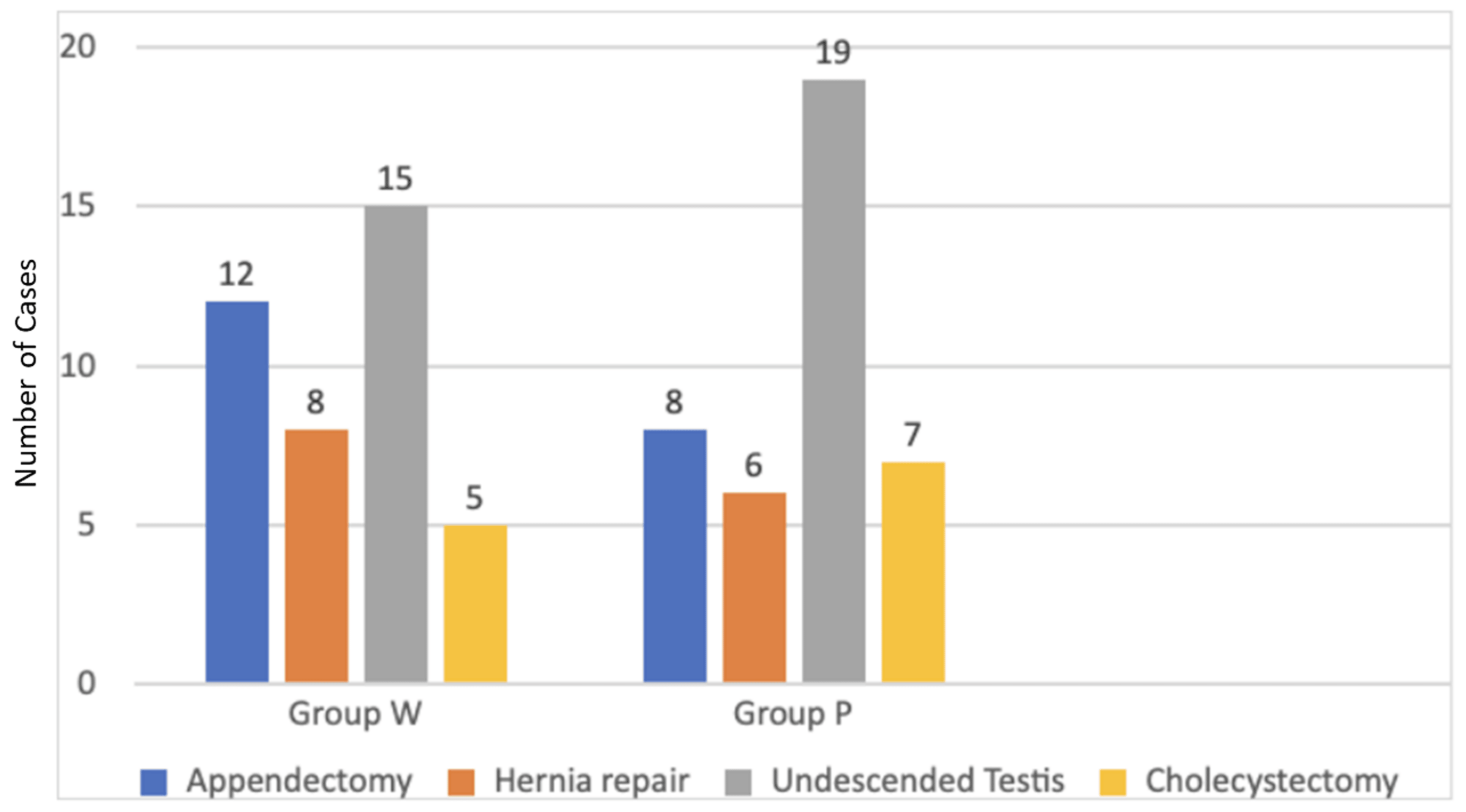
Comparison of operative procedures in both groups. Group P: intraperitoneal instillation of bupivacaine; Group W: trocar site infiltration of bupivacaine

The distribution of laparoscopic operative procedures is shown in Figure 2. Among these, orchidopexy, performed for undescended testes in male patients, was the most common procedure across both groups, followed by appendectomies, hernia repair, and cholecystectomy.

Table 1 comprehensively compares key outcomes between Group P and Group W, including mean pain scores and highlighting statistically significant differences (p-value=0.03). Pain scores were measured at intervals of one, two, four, six, and eight hours postoperatively in both groups. Group P consistently reported lower pain scores at all assessment intervals compared to Group W. Statistical analysis revealed significant differences in pain scores between the two groups at each time point (all p-values < 0.05), indicating that the intervention administered to Group P was associated with a more pronounced reduction in pain compared to Group W throughout the study period. Pronounced differences in pain scores were observed at both two and four hours postoperatively, with the most notable difference at the two-hour mark (p < 0.001), where a t-value of 4.46 was recorded. After six hours, pain scores were similar in both groups. Throughout all time intervals, Group P consistently demonstrated lower pain scores, indicating that intraperitoneal instillation of bupivacaine is more effective than trocar site infiltration for reducing postoperative pain in our study. 

**Table 1:** Comparison of mean pain scores, calculated pain scores at measured intervals, and secondary outcomes between the two groups * p-value significant at p<0.05, ** p-value significant at p <0.001 Statistical analysis was performed using the independent samples t-test, with the corresponding t-values and p-values displayed in the second last, and last column respectively. Group P: intraperitoneal instillation of bupivacaine; Group W: trocar site infiltration of bupivacaine

	Group W (n=40)	Group P (n=40)	t-value	p-value
Mean pain score, mean±SD	19.15±3.8	17.30±4.0	5.19	0.03*
Interval pain scores, mean±SD				
Pain score at 1 hours	2.70±1.32	2.10±1.28	2.06	0.04*
Pain score at 2 hours	4.35±1.62	3.01±1.01	4.46	<0.001**
Pain score at 4 hours	5.25±1.90	3.85±1.59	3.56	0.001**
Pain score at 6 hours	4.91±1.97	4.10±1.28	2.15	0.03*
Pain score at 8 hours	3.75±1.93	3.01±1.10	2.13	0.04*
Secondary outcomes, n (%)				
Need for rescue analgesia	17 (42.5%)	4 (10%)	-	0.001**
Incidence of shoulder pain	22 (55%)	13 (32.5%)	-	0.04*

Additionally, secondary outcomes for both groups were evaluated, providing a holistic view of the comparative analysis. The incidence of shoulder pain was observed in both groups, with 42.5% of children in the W Group developing shoulder pain compared to only 10% in the P Group. This difference was statistically significant (p-value=0.04). Furthermore, four patients in the P Group and 17 in the W Group required rescue analgesia, with the P Group showing a statistically significant reduction in analgesic need (p=0.001).

No adverse postoperative events or complications were observed as a result of the surgical intervention or the medications administered.

## Discussion

Postoperative pain is multifactorial in origin, and all possible measures should be taken to prevent its occurrence through preemptive analgesia during surgery [12]. Underestimating the incidence and severity of postoperative pain in the pediatric population can lead to suboptimal treatment, which, in turn, may have long-term negative impacts on their behavior and emotions [13]. Pharmacological management using nonsteroidal anti-inflammatory drugs (NSAIDs) and narcotic analgesics is often associated with drug- and dose-specific side effects, emphasizing the need to explore other multimodal analgesia options during surgery [14]. The peritoneum, composed of a dual layer of mesothelial cells, plays a vital role in normal physiology and pathological conditions due to its secretory and absorptive capabilities [15]. Its dynamic absorptive properties, mediated by specific transport receptors, can be harnessed for drug absorption to reduce pain intensity or occurrence, allowing direct action at the site of pain origin in the abdominal cavity [16]. Local wound infiltration and intraperitoneal instillation using local anesthetic agents are documented approaches for managing postoperative pain in adults, but limited data exist regarding their use in the pediatric population [17].

Intraperitoneal instillation of bupivacaine dampens the irritating effects of pneumoperitoneum by reducing the local inflammatory response, desensitizing peritoneal nociceptors, and preventing central sensitization through direct modulation of visceral pain at the site of surgical injury or irritation. In contrast, local trocar site infiltration primarily addresses the somatic component of postoperative pain. We opted to use the FACES Pain Scale for pain assessment due to its child-friendly design, ease of understanding, and accurate self-reporting capability. This scale reduces anxiety in children by using pictures instead of numbers or complex scales, offers consistent and reliable pain measurement, and actively involves children in the pain assessment process [11,18].

Our study found no statistically significant differences in demographic or operative details between the two groups. However, the mean pain score was significantly lower in the intraperitoneal instillation group compared to the wound infiltration group. A recent study by Ergun et al. evaluated the role of intraperitoneal bupivacaine instillation in pediatric laparoscopic appendectomy [19]. They observed lower pain scores and reduced rescue analgesia requirements in patients who received intraperitoneal bupivacaine at the end of surgery. However, they did not directly compare trocar site bupivacaine with intraperitoneal bupivacaine, as our study did. Another study by Di Pace et al. demonstrated that intraperitoneal ropivacaine significantly reduced the visceral component of post-laparoscopy pain in children, which is the primary type of pain in the postoperative period [20]. They also reported that local trocar infiltration had a limited role in reducing parietal incisional pain, highlighting the superiority of intraperitoneal instillation of local anesthetic agents. Similarly, Yong and Guang found intraperitoneal instillation of local anesthetics to be the most effective method for pain control, suggesting its integration into enhanced recovery pathways after laparoscopic surgery [21].

In another study, the effectiveness of intraperitoneal bupivacaine was compared with saline instillation in pediatric laparoscopic surgery [22]. The bupivacaine group demonstrated lower pain scores, better hemodynamic profiles, and reduced postoperative analgesia requirements. However, this study used xylocaine wound infiltration in both groups. In contrast, our study used the same drug for both wound infiltration and peritoneal instillation, thereby eliminating the confounding effects of different pharmacokinetics on postoperative pain. Additionally, we opted to compare wound infiltration with peritoneal instillation rather than saline instillation to determine the most effective method for pain control in pediatric laparoscopic surgery. A systematic review by Hamill et al. on the use of intraperitoneal local anesthetics in pediatric laparoscopic surgery concluded that this approach significantly reduces postoperative pain scores, the need for rescue analgesia, and the reliance on opioids, potentially avoiding opioid-associated complications in the pediatric population [23].

Our study also compared secondary pain outcomes in both groups, including the incidence of shoulder pain and the need for rescue analgesia postoperatively. Ergun et al., in their study, evaluated shoulder pain at one, six, 12, and 24 hours [19]. They observed that the group receiving bupivacaine had a significantly lower incidence of shoulder pain after 12 and 24 hours (p-value < 0.05). Similarly, shoulder pain was observed in both groups in our study. However, as both groups received bupivacaine, the incidence of shoulder pain was notably lower in the intraperitoneal instillation group (32.5%) compared to the trocar site infiltration group (55%). This difference was statistically significant. Additionally, we assessed the need for rescue analgesia in both groups. A similar comparison was conducted by Karnik and colleagues, who evaluated the use of ultrasound-guided transversus abdominis plane (TAP) bupivacaine block versus local infiltration during pediatric laparoscopic surgeries [24]. In their study, the TAP block group had a significantly lower need for rescue analgesia (8/46) compared to the local infiltration group (30/46; P<0.001). Our findings closely align with these results. Patients in our study who received peritoneal instillation of bupivacaine showed a lower need for rescue analgesia (4/40) compared to those in the local wound infiltration group (17/40; P<0.001).

Limitations

One of the limitations of our study was the small sample size which restricts the generalizability of our findings, highlighting the need for larger, multicenter trials to validate these results. Secondly, the absence of a placebo control group (e.g., saline instillation) in our study design limits our ability to assess the comparative efficacy of the techniques. Lastly, we did not investigate long-term outcomes, such as the incidence of chronic pain, which would offer a more comprehensive understanding of the benefits of intraperitoneal bupivacaine instillation

## Conclusions

Intraperitoneal instillation of bupivacaine provided more effective overall pain management compared to trocar site infiltration in pediatric laparoscopic procedures, likely due to its broader coverage of peritoneal pain sources. This technique involves the direct instillation of the analgesic into the peritoneal cavity at the surgical site, facilitating absorption and potentially enhancing postoperative pain relief while reducing reliance on systemic analgesics. The results indicate that intraperitoneal instillation improves postoperative pain control, which may facilitate earlier mobilization and enhance overall patient comfort. This approach could further optimize the benefits of minimally invasive surgery in the pediatric population by prioritizing effective pain management. These findings suggest that intraperitoneal analgesia may be a valuable approach for postoperative pain management in pediatric laparoscopic procedures, warranting further investigation and broader clinical evaluation.
